# An *In vitro* assessment of the genotoxic potential of short-, medium-, and long-chain triacylglycerides

**DOI:** 10.3389/ftox.2026.1807786

**Published:** 2026-04-08

**Authors:** Sydney Schreppler, Nikifar Lazouski, Lars Damen, Roy Hoffmans, Dejan Petrovic, Kathleen C. Alexander

**Affiliations:** 1 Savor Foods Limited, San Jose, CA, United States; 2 Charles River Laboratories Den Bosch B.V., Hertogenbosch, Netherlands

**Keywords:** genotoxicity, long-chain triacylglycerides, medium-chain triacylglycerides, mutagenicity, saturated fatty acids, short-chain triacylglycerides, synthetic fat, triacylglycerides

## Abstract

The genotoxic potential of a mixture of short-, medium-, and long-chain triacylglycerides (SMLCT), a novel synthetic fat intended as a non-animal alternative for food applications, was evaluated using a standard battery of *in vitro* assays in accordance with OECD Test Guidelines. Studies included a bacterial reverse mutation assay (OECD Test Guideline 471) with *Salmonella* Typhimurium and *Escherichia coli* strains at concentrations up to 5000 µg/plate; an *in vitro* mammalian gene mutation test in L5178Y mouse lymphoma cells (OECD Test Guideline 490) at concentrations up to 31 μg/mL; and an *in vitro* micronucleus assay in cultured human peripheral blood lymphocytes (OECD Test Guideline 487) at concentrations up to 7.8 μg/mL. Across all assays, SMLCT did not induce biologically relevant increases in revertant colonies, mutant frequencies, or micronucleated binucleated cells. These findings indicate that SMLCT is neither mutagenic nor clastogenic under the conditions of the studies, supporting its safety for use as a dietary fat ingredient in finished food.

## 1 Introduction

Dietary fat is a source of essential fatty acids and fat-soluble vitamins, and a critical component of a healthy diet. It is consumed primarily as triacylglycerides (TAGs) but may contain other constituents such as mono- and diglycerides, phospholipids, free fatty acids, tocopherols, and sterols. The nutritional value and biological effects of triacylglycerides are principally determined by their fatty acid composition. Structurally, TAGs consist of a glycerol backbone esterified with three fatty acids, the chain length and degree of unsaturation of which can vary widely, with the melting point of the fat or oil increasing proportionally to the degree of saturation (Patterson, 2009; Hidalgo et al., 2016). Dietary even-chain TAGs are incorporated into chylomicrons that are distributed systemically to provide tissues with a source of essential fatty acids which are required for cell membrane maintenance, hormone synthesis and signaling, and absorption of fat-soluble vitamins, and which ultimately function as a calorie-dense energy source (Maki et al., 2021; Wang et al., 2023; Zhao et al., 2024). Short-chain and smaller medium-chain saturated fatty acids (<C10:0) bypass this metabolic route and are subject to epithelial absorption without re-esterification and elicit a range of metabolic and immune signaling functions in addition to providing a dietary energy source (Schönfeld and Wojtczak, 2016; Rekha et al., 2024). Dietary odd-chain TAGs are subject to the same lipase and β-oxidation to yield acetyl–coenzyme A (CoA) units, but yield a propionyl-CoA final product. Propionyl-CoA is subsequently metabolized to succinate-CoA for incorporation into the citric acid cycle; no evidence of toxicity from consumption of odd-chain fatty acid TAGs has been reported in the literature (Kurotani et al., 2017; Venn-Watson et al., 2020).

Based on the considerable history of consumption from both unmodified natural sources and commercial industrial production, TAGs and their component fatty acids do not generally present toxicological risk as part of the human diet (Bach and Babayan, 1982; Traul et al., 2000; Matulka et al., 2009; Zhou et al., 2017; Jadhav and Annapure, 2021; Maki et al., 2021; Wang et al., 2023; Hartwig, 2024; Zhao et al., 2024). Consumption of non-animal fats containing mono- and polyunsaturated fatty acids is generally accepted as having a lower risk of adverse cardiovascular outcomes compared with consumption of animal-sourced fats predominantly containing long-chain saturated and monounsaturated fatty acids (Guasch-Ferre et al., 2019; Kim et al., 2019; Neuenschwander et al., 2023; Zhao et al., 2024). Fats (and proteins) from alternative sources are also associated with lower agricultural land use, water consumption, and carbon emissions (Smetana et al., 2023; Mandouri et al., 2025). However, significant processing, chemical modification, and/or addition of excipients and stabilizers are typically required to generate non-animal fat products with textures and flavors comparable to animal-based ones (Dreher et al., 2020; Puşcaş et al., 2020; Sha and Xiong, 2020; Dreher et al., 2021; McClements and Grossmann, 2021).

The ever-increasing global food supply demand requires that innovative, novel sources of dietary fat be developed to provide safe, nutritionally sound fat ingredients for use in food. In order to address the environmental impact of producing the plant-based dietary fat required to meet the ever-increasing demand for fat macronutrient ingredients, the food industry has, over the last 4 decades, continued to develop innovative dietary fat and fat replacement ingredients.

The novel synthetic dietary fat ingredient—short-, medium-, and long-chain triacylglycerides (SMLCT)—is a blend of TAGs comprised of an equal abundance of odd-chain and even-chain saturated fatty acids. Individual fatty acids with chain lengths ranging from C4 to C22 are present in an approximately consistent distribution, with the highest relative concentrations observed for lauric and tridecyclic acids (C12:0 and C13:0, respectively), each accounting for approximately 6%–12%. The greatest overall abundance lies between caprylic acid (C8:0; 3%–12%) and margaric acid (C17:0; 5%–10%). Lower levels (<5%) are observed for short-chain fatty acids as well as stearic (18:0), nonadecylic (C19:0), arachidic (C20:0), heneicosylic (C21:0), and behenic (C22:0) acids. Typically, odd-chain fatty acids are not abundant within TAGs present in dietary fats and are a minor class of dietary fatty acids. The metabolization of TAGs with odd-chain fatty acids generates products distinct from their even-chain counterparts, notably propionyl-CoA instead of acetyl-CoA. Several metabolic steps are necessary to yield succinyl-CoA, which is incorporated into the citric acid cycle (Nelson and Cox, 2004; Gotoh et al., 2008). Findings of metabolic studies investigating odd-chain fatty acids as dietary fat indicate that consumption of pentadecanoic and heptadecanoic acids in adult human subjects have a significant positive impact on serum adipokines (leptin, plasminogen activator inhibitor-1, and visfatin; *p* < 0.05) (Kurotani et al., 2017).

SMLCT is intended for use as an alternative to animal- and plant-derived solid fats in finished foods and could serve to address the limitations of non-animal sources with sufficient physiochemical character, and does not have the agricultural or environmental impacts associated with production of plant-based fats. SMLCT, as a dietary fat ingredient, is produced by a thermochemical synthetic method which yields an approximately standard distribution of TAG fatty acid chain-lengths between C4 and C22 (Table 1).

**TABLE 1:** Product specification: principal components and distribution of fatty acids in SMLCT.

Parameters	Specification	Result	Method of analysis
Physical state at 25 °C	Solid to liquid	Pass	Visual
Color	White to off-white (solid state); colorless to yellow (liquid state)	Pass	Visual
Acid value (mg KOH/g)	<2	0.9	AOCS Ca 5a-40
Hydroxyl value (mg KOH/g)	<15	6.9	USP 401 (2017), potentiometric titration
Iodine value (mg I_2_/100 g)	<4	<1.0	ISO 3961 (2018), iodometric titration
Soap value (ppm)	<100	<1	NGD C8-1976
Peroxide value (mEq O_2_/kg)	<5	1.2	AOCS Cd 8-53
P-anisidine (AnV)	<10	0.08	AOCS Cd 18-90
TBA value (mcg/g)	<1	<1.00	AOCS Cd 19-90
Isoacids (%)	<5	2.81	ISO_FAME (AOCS Ce 1a-13, Ce 1h-05)^a^
SMLCT distribution of fatty acids			
Butanoic acid (C4) (%)	<5	<0.06​	FALT_S (996.06 AOAC and AOCS Ce 1h-05)^b^
Pentanoic acid (C5) (%)	<5	0.33​	
Hexanoic (caproic) acid (C6) (%)	<5	1.27​	
Heptanoic acid (C7) (%)	<5	2.75​	
Octanoic (caprylic) acid (C8) (%)	3–12	4.25​	
Nonanoic acid (C9) (%)	3–12	5.49​	
Decanoic (capric) acid (C10) (%)	3–12	6.26​	
Undecanoic acid (C11) (%)	3–12	7.01​	
Dodecanoic (lauric) acid (C12) (%)	6–12	7.56​	
Tridecanoic acid (C13) (%)	6–12	7.97​	
Tetradecanoic (myristic) acid (C14) (%)	5–12	8.18​	
Pentadecanoic acid (C15) (%)	5–12	8.18​	
Hexadecanoic (palmitic) acid (C16) (%)	5–10	8.26​	
Heptadecanoic acid (C17) (%)	5–10	7.33​	
Octadecanoic (stearic) acid (C18) (%)	<7	5.95​	
Nonadecanoic (C19) (%)	<5	3.78​	
Icosanoic (arachidic), henicosanoic, and docosanoic (behenic) acid (C20–C22) (%)	<5	<2.76​	
Heavy metals			
Arsenic (ppm)	≤0.05	<0.01	ICP-MS
Cadmium (ppm)	≤0.1	<0.005	
Lead (ppm)	≤0.1	<0.005	
Mercury (ppm)	≤0.05	<0.005	
Microbial parameters			
Aerobic plate count (CFU/g)	≤10	<10	AOAC OMA 990.12
Total coliforms (CFU/g)	≤10	<10	AOAC OMA 991.14
Yeast (CFU/g)	<10	<10	AOAC OMA 2014.05
Mold (CFU/g)	<10	<10	AOAC OMA 2014.05

AnV = anisidine value; AOAC, association of official analytical chemists; AOCS, American Oil Chemists’ Society; CFU, colony-forming units; FAME, fatty acid methyl esters; I_2_ = iodine; ICP-MS, inductively coupled plasma mass spectrometry; ISO, international organization for standardization; KOH, potassium hydroxide; mcg = micrograms; mEq = milliequivalent; O_2_ = oxygen; OMA, official methods of analysis; ppm = parts per million; SMLCT, short-, medium, and long-chain triacylglycerides; USP, united states pharmacopeia.

^a^ ISO_FAME, is an internal method based on AOCS, Ce 1a-13 and Ce 1h-05.

^b^ FALT_S is a custom validated method from Eurofins, mostly based on 996.06 AOAC, and AOCS, Ce 1h-05.

The oral toxicity and genotoxicity of most TAGs comprising SMLCT has been evaluated and is reported in the literature. Matulka et al. (2021) reported an absence of mutagenic potential in a genotoxicity test battery similar to that reported herein. The algal oil rich in the odd-chain fatty acids pentadecanoic acid (C15:0) and heptadecanoic acid (C17:0) evaluated by Matulka et al. (2021) did not induce an increase in revertant colonies of *Salmonella* Typhimurium strains in a bacterial reverse mutation assay up to 3,000 and 5000 µg/plate in a main test and in confirmatory tests, respectively. The authors also reported no chromosomal aberrations in bone marrow cells of rats administered algal oil at up to 2000 mg/kg body weight for 24 or 48 h. Mitotic indices were significantly increased at 24 h in females administered 1500 mg/kg body weight and males administered 2000 mg/kg body weight, but not in males administered 1500 mg/kg body weight or females administered 2000 mg/kg body weight. At 48 h post-administration, mitotic indices were not significantly different in any treatment group (Matulka et al., 2021). While these data confirm the absence of genotoxic potential for medium odd-chain fatty acids, the genotoxicity of odd-chain fatty acid TAGs of shorter chain lengths (C5:0 to C11:0) in a heterogeneous mixture similar to SMLCT has not been tested directly. The composition and production process of SMLCT are novel, and the safety of this synthetic fat product for human consumption has yet to be directly established; therefore, a battery of genetic toxicity studies represents an appropriate first step.

The studies reported in this article were designed to evaluate the genotoxic potential of SMLCT in accordance with Organisation for Economic Co-operation and Development (OECD) Test Guidelines 471, 490, and 487 for the bacterial mutation assay (OECD, 2020), *in vitro* mammalian cell mutation assay (OECD, 2016), and *in vitro* micronucleus assay in human lymphocytes, respectively (OECD, 2023).

## 2 Materials and methods

The studies described herein were conducted by Charles River Laboratories in the Netherlands according to the OECD principles of Good Laboratory Practice (GLP), as accepted by regulatory authorities throughout the European Union; the United States (Food and Drug Administration and Environmental Protection Agency); Japan (Ministry of Health, Labour and Welfare; Ministry of Agriculture, Forestry and Fisheries; and Ministry of Economy, Trade and Industry); and other countries that are signatories to the OECD Mutual Acceptance of Data Agreement. The standard battery of toxicological tests conducted to assess the genotoxic and mutagenic potential of SMLCT included a bacterial reverse mutation assay (OECD Test Guideline 471) at doses of 5–5000 µg/plate, an *in vitro* mammalian gene mutation test in 5178Y mouse lymphoma cells (OECD Test Guideline 490) at concentrations of up to 31 μg/mL, and an *in vitro* micronucleus test in human peripheral blood lymphocyte at concentrations up to 7.8 μg/mL (OECD Test Guideline 474) (OECD, 2016; OECD, 2020; OECD, 2023).

### 2.1 Materials

The SMLCT (Lot EST009) test article was provided by Savor Foods Ltd. (California, United States). SMLCT is a white to off-white soft solid TAG product synthesized via a thermochemical process in which fatty acids are produced by oxidation of non-petroleum-based paraffins. The resulting fatty acids are purified and esterified with glycerol to produce a TAG product containing short-, medium-, and long-chain saturated fatty acids with carbon chain-lengths ranging from C4 to C22 (Table 1). Analysis included measures of acid, hydroxyl, iodine, soap, peroxide, isoacids, and p-anisidine values which were all within the set specifications. SMLCT was shown to be soluble in tetrahydrofuran (THF) following a series of solubility tests in several organic solvents including ethanol, dimethyl sulfoxide (DMSO), and n-hexane, among others. SMLCT was soluble in THF at 400 mg/mL and below. Furthermore, a dose formulation analysis and stability assessment of SMLCT was conducted, demonstrating homogeneity at intermediate and high concentrations, with a coefficient of variation of 20%.

### 2.2 Methods

#### 2.2.1 Bacterial reverse mutation assay

The bacterial reverse mutation assay was conducted in accordance with OECD Test Guideline 471 (OECD, 2020) and principles of GLP from OECD (OECD, 1998) and other appropriate authorities.

SMLCT dissolved in THF (BioSolve BV; Valkenswaard, the Netherlands) was evaluated for mutagenic potential; strains of *Salmonella* Typhimurium (TA100 and TA1535) and *Escherichia coli* (WP2 *uvrA*) were used for the detection of point mutations, and *S.* Typhimurium strains TA98 and TA1537 were employed for detection of frameshift mutations. The microbial test strains were obtained from Trinova Biochem GmbH (Germany) and The National Collections of Industrial and Marine Bacteria (Aberdeen, United Kingdom). Spontaneous revertant colonies in response to treatment with SMLCT were determined using the Maron and Ames (Maron and Ames, 1983) method also described in OECD Test Guideline 471 (OECD, 2020).

The positive control chemicals selected according to test guidelines were as follows: sodium azide, 2-nitrofluorene, 2-aminoanthracene (2-AA), methyl methanesulfonate (MMS), and 4-nitroquinoline *N*-oxide. These compounds generally serve as strain-specific inducers of mutagenicity. Also, while 2-AA requires metabolic activation to induce mutagenicity, the remaining compounds are direct mutagens in the respective target bacterial strains. Physiological saline (Merck; Darmstadt, Germany) was used as vehicle for sodium azide, and the remaining positive controls were dissolved in DMSO (Merck; Darmstadt, Germany). Unless otherwise stated above, all reagents were supplied by Sigma (Germany). The metabolic activation system, compliant with OECD Test Guideline 471 (OECD, 2020), was a commercial S9 liver fraction and Cofactor A supplied by Trinova Biochem GmbH (Giessen, Germany). The S9 fraction (Lot Nos. 4687, 4694, 4713, and 4777) was prepared from male Sprague Dawley rats dosed orally with a suspension of phenobarbital (80 mg/kg body weight) and ß-naphthoflavone (100 mg/kg). Each S9 batch was characterized with the mutagens benzo-(a)-pyrene and 2-AA, which require metabolic activation in tester strain TA100.

The study was conducted using the plate-incorporation method described by OECD Test Guideline 471 (OECD, 2020) for bacterial reverse mutation assay. The negative control (THF) and relevant positive controls were concurrently tested in each strain in the presence and absence of S9 mix. Top agar (Oxoid Ltd.) was melted by heating to 45 °C ± 2 °C; to 3 mL of molten top agar were added 0.1 mL of fresh bacterial culture (10^9^ cells/mL) of the tester strains, 0.05 mL of a dilution of the test material in THF or 0.1 mL of a solution of the control solution and either 0.5 mL S9 mix or 0.5 mL 0.1 M phosphate buffer (Merck; Germany—for assays without metabolic activation). The contents of the top agar tube were vortex-mixed and poured onto a selective agar plate. Once the top agar solidified, plates were inverted and incubated in the dark at 37.0 °C ± 1.0 °C for 48 ± 4 h following which revertant colonies (histidine-independent for *S.* Typhimurium and tryptophan-independent for *E. coli*) were counted using the automated Sorcerer Colony Counter. Plates with precipitates of test materials were counted manually.

Three separate experiments were conducted: one dose range–finding study and two main studies using the pre-incubation method described above. The dose range–finding study was used to determine a reasonable upper test concentration of SMLCT and was conducted in the presence or absence of S9 mix, using one plate/concentration. In the first main study, concentrations of 1.7, 5.4, 17, 52, 164, 512, 1600, and 5000 μg/plate were tested in triplicates (OECD Test Guideline 471) (OECD, 2020). Due to the precipitation of test materials at 1600 and 5000 μg/plate, the second main experiment was conducted on all tester strains with 48, 86, 154, 275, 492, and 878 μg/plate in triplicates. A third experiment (confirmatory test) was conducted only on strain TA1537 in the absence of S9 mix at concentrations of 10, 100, 200, 300, 400, and 500 μg/plate. Toxicity was determined by a reduction of the background lawn and/or a decrease in the number of revertant colonies. Individual plates were counted for revertant colonies. The average and standard deviation of the number of revertant colonies were calculated based on the individual results of three replicates. Data interpretation followed the acceptability criteria set out by the testing lab and described in Levy et al. (2019), in accordance with OECD Test Guideline 471.

#### 2.2.2 *In Vitro* micronucleus assay in cultured peripheral human lymphocytes

The *in vitro* micronucleus assay was conducted in accordance with OECD Test Guideline 487 (OECD, 2023) and assessed the ability of SMLCT to induce micronuclei in cultured human lymphocytes, an indicator of clastogenicity and/or aneugenicity.

Cultured peripheral human lymphocytes were used as the test system as recommended by OECD Test Guideline 487. Blood samples were collected from healthy, non-smoking volunteer adults (ages 18–35 years). Collection was conducted by venipuncture into sterile bottles containing sodium heparin (Vacuette, Greiner Bio-One; Alphen aan den Rijn, the Netherlands). Heparinized whole blood was cultured in 5 mL or 4.8 mL culture medium (Life Technologies) to which phytohemagglutinin (Remel Europe Ltd.; Dartford, United Kingdom) was added in the presence and absence of S9 mix. Cells were cultured under optimal conditions (26%–97% humidity, 5.0% ± 0.5% CO2, and 37.0 °C ± 1.0 °C). As described above, the metabolic activation system comprised the S9 fraction prepared from male Sprague Dawley rats dosed orally with a suspension of phenobarbital (80 mg/kg body weight) and ß-naphthoflavone (100 mg/kg).

Lymphocytes (derived from 0.4 mL blood) from a healthy donor were added to 4.8 mL or 5 mL culture medium with and without metabolic activation, respectively, to which 0.1 mL (9 mg/mL) phytohemagglutinin was added. Cells were cultured for 48 ± 2 h, following which predetermined concentrations of SMLCT were added and incubated for 3 h and 24 h in the absence of S9 mix, or for 3 h in the presence of S9 mix. Cytochalasin B (Sigma) was added to the cells simultaneously with the test material at the 24-h exposure time. A negative control was included at each exposure time. Lymphocytes were cultured in duplicate for the 3-h incubation time along with appropriate positive controls. Following exposure to the test material, cells were separated from the culture medium by centrifugation, washed with Hanks’ Balanced Salt Solution (HBSS; Life Technologies, Paisley; United Kingdom), resuspended in cytochalasin B (5 μg/mL), and incubated for another 24 h in the absence of S9 mix. In all experiments, there were concurrent positive and negative controls. The negative control was THF, the solvent in which SMLCT was dissolved. The positive controls were (1) mitomycin C (Lot No. SLCJ2007; Sigma, Germany), a direct-acting clastogen; (2) colchicine (Lot No. A0417355; Acros Organics; Belgium), a direct acting aneugen used in the absence of metabolic activation; and (3) cyclophosphamide (Lot No. A0437827; Acros Organics; Belgium), an indirect-acting clastogen requiring metabolic activation.

Cells were thereafter fixed in acetic acid (Merck; Germany). The cytotoxicity of SMLCT on human lymphocytes was determined using the cytokinesis-block proliferation index method. To prepare slides, cells in the remaining pellets were resuspended in 1% Pluronic F68 (Applichem; Darmstadt, Germany) and then centrifuged. The cells were subsequently swollen in a hypotonic (0.56% w/v) potassium chloride solution (Merck; Germany). Fixed cells were placed on appropriately identified slides, allowed to dry, and stained with 6.7% (v/v) Giemsa (Merck; Germany), and the slides were mounted with coverslips. On each slide, a minimum of 1000 binucleated cells were examined using a light microscope. Binucleated cells were scored using criteria published by Fenech (1996), Kirsch-Volders et al. (2000), and Fenech et al. (2003). The data were deemed acceptable if they complied with predetermined criteria for positive and negative controls, and if there was a statistically significant increase in the number of binucleated cells compared with the negative control, and if colchicine induced a statistically significant increase in the number of binucleated cells with micronuclei in at least one experiment. Statistical analysis was conducted using GraphPad Prism Version 8.4 (GraphPad Software; San Diego, United States), and this was used to determine positive response (clastogenicity or aneugenicity) in response to cell exposure to SMLCT. The test substance was deemed clastogenic under the following conditions: (a) at least one of the test concentrations had a statistically significant increase (Fisher’s exact test, one-sided, *p* < 0.05) in the number of binucleated cells with micronuclei; (b) the increase in the number of binucleated cells with micronuclei was dose-related in at least one experimental condition when evaluated with the Cochran Armitage trend test; and (c) the result obtained under the conditions above was outside the 95% historical control limit.

The experiments were conducted in 2 phases: a dose range–finding test with the first cytogenetic test, and a second cytogenetic assay. The dose range–finding study was conducted to select the appropriate concentrations for the *in vitro* micronucleus test. For the dose range–finding test, lymphocytes (0.4 mL blood, as described above) were cultured for 48 ± 2 h and, based on the results of the dose range–finding study, were exposed to 0.24, 0.49, 0.97, 2.0, 3.9, or 7.8 μg test SMLCT/mL culture medium for 24 h in the absence of S9 mix. The first cytogenetic assay was conducted in duplicates at 2.0, 3.9, and 7.8 μg SMLCT/mL culture medium with and without S9 mix for 3 h. In order to assess the possible clastogenicity and aneugenicity of SMLCT, a second cytogenetic study was conducted in which a 48-h human lymphocyte culture was exposed to the SMLCT for 24 h without S9 mix.

#### 2.2.3 *In Vitro* mammalian gene mutation test in 5178Y mouse lymphoma cells

This study, conducted in accordance with OECD Test Guidance 490 (OECD, 2016), investigated the mutagenic potential of SMLCT via its ability to induce forward mutations at the thymidine kinase (TK) locus in L5178Y mouse lymphoma cells, either in the absence or presence of a metabolic activation system (S9 mix). This system detects a broad range of mutations including base pair alterations, frame shift mutations, small deletions, and clastogenic effects.

The test system employed in the study was the L5178Y mouse lymphoma cell line which is sensitive to the mutagenic activity of a broad range of chemical classes (OECD, 2016), and substances which test positive in this assay are presumed to be a potential mammalian cell mutagen. The test system, L5178Y/TK^+/−^3.7.2C mouse lymphoma cells, was sourced from the American Type Culture Collection (United States). Cell stock cultures stored in ultra-low freezers maintained at −150 °C and which tested negative for *mycoplasma* contamination were thawed and grown in a basic cell-culture medium consisting RPMI 1640 HEPES-buffered medium (Dutch modification) containing penicillin/streptomycin (50 U/mL and 50 μg/mL, respectively), 1 mM sodium pyruvate, and 2 mM L-glutamine supplemented with 10% heat-inactivated horse serum. The following media were used depending on the desired growth condition: exposure medium consisting of basic medium supplemented with 5%–10% (v/v) heat-inactivated horse serum; selective medium consisting of basic medium supplemented with 20% (v/v) heat-inactivated horse serum and 5 μg/mL trifluorothymidine (TFT); or non-selective medium consisting of basic medium supplemented with 20% (v/v) heat-inactivated horse serum. Cultured cells were maintained at 80%–100% humidity, 5.0% ± 0.5% CO_2_, and 37.0 °C ± 1.0 °C. As described for the other studies above, the metabolic activation system comprised S9 fraction prepared from male Sprague Dawley rats dosed orally with a suspension of phenobarbital (80 mg/kg body weight) and ß-naphthoflavone (100 mg/kg).

The first step in the study was the cleansing of the cells in which the mouse lymphoma cells were grown for 24 h in growth media supplemented with hypoxanthine (10^−4^ M), aminopterin (2 × 10^−7^ M), and thymidine (1.6 × 10^−5^ M) to reduce the occurrence of spontaneous mutants. This was followed by a recovery period of 48 h in medium containing hypoxanthine and thymidine only. Thereafter, cells were grown in growth medium for at least 24 h before initiation of experiments. A 30-mL centrifuge tube was seeded with cells at a minimum density of 8 × 10^6^ cells/tube for the 3-h treatment phase, or 6 × 10^6^ cells/tube for the 24-h treatment phase. Cells were kept in kept in shaking incubator maintained at 37.0 °C ± 1.0 °C. In the 3-h treatment phase, cells were exposed to SMLCT (dissolved in THF) in the presence and absence of S9 mix following which cells were separated by a centrifugation step and resuspended in HBSS. Cells were centrifuged and resuspended in 50 mL of growth medium. The 24-h treatment phase was conducted as in the 3-h period above, except exposure took place in the absence of metabolic activation and cells were resuspended at 20 mL in growth medium. Cell suspensions were counted with a Coulter particle counter.

For the expression of mutant phenotype, 2 days after incubation with SMLCT, previously treated cells were subcultured daily to maintain log-phase growth. Cells were thereafter plated for the determination of cloning efficiency on Day 2 (CE_Day2_) and mutation frequency (MF). To determine CE_Day2_, cells were seeded into the wells of two 96-well microtiter plates per SMLCT concentration in non-selective medium. To determine MF, a total of 9.6 × 10^5^ cells/concentration were suspended in five 96-well microtiter plates in the selective TFT medium. Microtiter plates were incubated for 10–12 days. Thereafter, plates for the selective TFT medium were stained for 1.5–2 h with 0.5 mL MTT (3-[4,5-dimethylthiazol-2-yl]-2,5-diphenyltetrazolium bromide), and each plate was scored for CE_Day2_ and MF using either the naked eye or a microscope. Cells were thereafter analyzed for the determination of mutant colonies in accordance with established protocol described in OECD (OECD, 2016) or protocols published by Clive et al. (1987), Clive and Spector (1975), Cole and Arett, 1984, Clive et al. (1995), and Moore et al. (1999). Data were analyzed for increases in MF and to assess the biological relevance of the response, including a comparison of the results with the historical control data range in accordance with established acceptability criteria.

The study was conducted in 2 phases: a dose range–finding test and mutagenicity test. Following a solubility assessment of the test substance, feasible doses for the mutagenicity study were determined by seeding 8 × 10^6^ cells or 6 × 10^6^ cells for 24-h treatment, suspended in the exposure medium at a range of 7.8–125 μg SMLCT/mL for three or 24 h in the absence of S9 mix and for 3 h in the presence of the S9 mix. Based on the result of the dose range–finding study, two mutagenicity tests were conducted: (1) presence and absence of the S9 mix at concentrations of 5, 7.5, 10, 12.5, 15, 20, 25, and 31 μg SMLCT/mL in the exposure medium for 3 h, and (2) absence of the S9 mix at concentrations of 3.9, 7.8, 16, and 31 μg SMLCT/mL in the exposure medium for 24 h. The concurrent positive controls were MMS (Sigma, Germany) for use without metabolic activation and cyclophosphamide (Life Technologies; Paisley, United Kingdom) for use with metabolic activation.

## 3 Results

### 3.1 Bacterial reverse mutation assay

The bacterial reverse mutagenicity (Ames) assay on SMLCT using *S.* Typhimurium strains TA1535, TA1537, TA98, TA100 and *E. coli* WP2 *uvrA*, both with and without metabolic activation (S9 mix), revealed no biologically relevant mutagenic activity across the three mutation experiments. Precipitation of the test material was observed at a concentration of 512 μg/plate and upwards in all tester strains in the absence and presence of S9 mix.

Cytotoxicity was not observed in any strain at any concentration aside from TA1535 at the top concentration, 5000 μg/plate, in the absence of S9 mix (Supplementary Table S1). In a second experiment, all strains exposed to concentrations of 48–878 μg/plate in the absence and presence of S9 mix demonstrated no cytotoxicity and no biologically relevant or dose-dependent increases in the number of revertant colonies. However, compared with the solvent control, strain TA1537 exhibited a 6-fold increase in the number of revertant colonies in the absence of the S9 mix at a concentration of 154 μg/plate (Table 2). These increases were not clearly dose-related, were within the historical control data range, and were related to a low number of revertants in the solvent control. To further assess the relevance of the result, a follow-up study was conducted with strain TA1537 using concentrations of 10, 100, 200, 300, 400, and 500 μg/plate; no precipitate formation, cytotoxicity, or biologically relevant increases in the number of revertant colonies were observed (Table 3).

**TABLE 2:** Mutagenicity testing of SMLCT in the *Salmonella* Typhimurium mutation assay and the *Escherichia coli* mutation assay with or without metabolic activation.

Concentration (µg/plate)	Revertant colonies per plate (mean ± SD)									
	*Salmonella* Typhimurium								*Escherichia coli*	
	TA98		TA100		TA1535		TA1537		WP2 *uvrA*	
	-S9	+S9	-S9	+S9	-S9	+S9	-S9	+S9	-S9	+S9
Vehicle control (THF)	17.7 ± 2.1	29.0 ± 6.0	127.0 ± 14.4	67.7 ± 3.5	14.0 ± 8.2	15.3 ± 4.0	2.0 ± 1.7	8.7 ± 3.5	29.3 ± 8.5	53.3 ± 11.2
48	24.7 ± 6.4	27.0 ± 1.0	73.0 ± 17.3	102.3 ± 19.1	11.0 ± 1.0	15.3 ± 7.6	8.3 ± 3.2	4.3 ± 1.5	32.0 ± 4.4	51.3 ± 9.0
86	27.0 ± 7.2	31.3 ± 7.5	59.7 ± 4.0	111.0 ± 17.3	13.7 ± 1.2	9.7 ± 1.5	9.0 ± 1.0	2.3 ± 1.2	31.3 ± 2.5	46.3 ± 10.0
154	24.7 ± 5.5	25.0 ± 4.4	61.7 ± 9.8	121.3 ± 23.4	10.7 ± 2.3	10.0 ± 2.6	12.0 ± 1.7	3.3 ± 3.5	24.7 ± 6.0	47.3 ± 8.5
275	31.0 ± 1.7	19.3 ± 4.2	67.3 ± 8.7	113.7 ± 8.4	13.0 ± 5.0	8.0 ± 1.0	5.7 ± 0.6	2.0 ± 1.0	29.0 ± 2.6	32.3 ± 14.6
492 ^a^	28.0 ± 6.1	19.3 ± 5.5	68.7 ± 12.5	131.7 ± 2.1	14.3 ± 4.0	7.7 ± 1.2	6.3 ± 3.8	5.0 ± 1.0	39.3 ± 8.5	31.3 ± 0.6
878 ^a^	16.0 ± 6.6	19.7 ± 3.2	63.0 ± 2.6	92.0 ± 11.0	11.7 ± 1.5	9.0 ± 1.0	5.0 ± 1.7	4.0 ± 1.0	24.0 ± 7.0	34.3 ± 8.1
Positive control ^b^ ^,^ ^c^	1917.7 ± 150.3	1304.0 ± 46.0	887.3 ± 70.6	1130.3 ± 129.9	968.3 ± 5.5	359.7 ± 128.7	923.0 ± 57.1	166.0 ± 85.9	1387.7 ± 173.0	354.0 ± 32.6

-S9 = in the absence of S9; +S9 = in the presence of S9; 2-AA, 2-aminoanthracene; 2-NF, 2-nitrofluorene; 4-NQO, 4-nitroquinoline *N*-oxide; AAN, 9-aminoacridine hydrochloride; ICR-191, acridine mutagen ICR-191; MMS, methyl methanesulfonate; SA, sodium azide; SD, standard deviation; SMLCT, short-, medium-, and long-chain triacylglycerides; THF, tetrahydrofuran.

^a^ The test material precipitated in the exposure medium.

^b^ Positive control -S9: TA98 = 10 µg/plate 2-NF; TA100 = 650 µg/plate MMS; TA1535 = 5 µg/plate SA; TA1537 = 2.5 µg/plate ICR-191; WP2 *uvrA* = 10 µg/plate 4-NQO.

^c^ Positive control + S9: TA98 = 1 µg/plate 2-AA; TA100 = 2 µg/plate 2-AA; TA1535 = 2.5 µg/plate 2-AA; TA1537 = 5 µg/plate AAN; WP2 *uvrA* = 15 µg/plate 2-AA.

**TABLE 3:** Confirmatory testing of the mutagenicity potential testing of SMLCT in the reverse mutation assay using *Salmonella* Typhimurium strain TA1537 in the absence of metabolic activation.

Concentration (µg/plate)	Revertant colonies per plate (mean ± SD)	
	*Salmonella* Typhimurium	
	TA1537	
	-S9	+S9
Vehicle control (THF) ^a^	5.3 ± 1.2	-
10	8.7 ± 3.8	-
100	11.7 ± 0.6	-
200	13.7 ± 5.8	-
300	6.3 ± 2.9	-
400	6.7 ± 2.1	-
500 ^a^	11.7 ± 5.1	-
2.5 (ICR-191, positive control)	683.0 ± 105.1	-

- = not reported; -S9 = in the absence of S9; +S9 = in the presence of S9; ICR-191, acridine mutagen ICR-191; MMS, methyl methanesulfonate; SD, standard deviation; SMLCT, short-, medium-, and long-chain triacylglycerides; THF, tetrahydrofuran.

^a^ The test material precipitated in the exposure medium.

Based on the study criteria, SMLCT was concluded to be non-mutagenic under the conditions of the bacterial reverse mutation Ames assay.

### 3.2 *In Vitro* micronucleus assay in cultured peripheral human lymphocytes

In the dose range–finding test and first cytogenetic assay, precipitation occurred at 7.8 μg/mL, with no significant pH changes, osmolality changes, or cytotoxicity. The numbers of binucleated cells with micronuclei observed in the positive and negative controls were within the respective ranges of historical data for the testing laboratory. The positive control chemicals mitomycin C, colchicine, and cyclophosphamide all produced a statistically significant increase in the numbers of binucleated cells with micronuclei. Conversely, in the first cytogenetic assay, SMLCT at all concentrations and exposure times (3 and 24 h) did not induce statistically significant or biologically relevant increases in the numbers of binucleated cells with micronuclei, either in the presence or absence of S9 mix (Table 4). Similarly, in the second cytogenetic assay, conducted to obtain more information on the clastogenicity and aneugenicity of SMLCT following a 24-h exposure in the absence of the S9 mix, no statistically significant or biologically relevant increases in the numbers of binucleated cells with micronuclei were observed (Table 4).

**TABLE 4:** Number of binucleated cells with micronuclei of human lymphocyte cultures treated with SMLCT in the first and second cytogenetic assays.

First assay without metabolic activation (-S9 mix); 3-h exposure time, 27-h harvest time				
Concentration (µg/mL)	Cytostasis (%)	Number of binucleated cells with micronuclei ^a^		
		1000	1000	2000
		A	B	A + B
0	0	1	2	3
2.0	8	2	1	3
3.9	15	1	2	3
7.8	27	1	5	6
0.20 MMC	50	30	26	56****
0.05 colchicine	39	9	11	20***
First assay with metabolic activation (+S9 mix); 3-h exposure time, 27-h harvest time				
0	0	2	1	3
2.0	4	2	0	2
3.9	0	1	4	5
7.8	5	0	2	2
7.5 CP	66	12	13	25****
Second assay without metabolic activation (-S9 mix); 24-h exposure time, 24-h harvest time				
0	0	5	3	8
2.0	11	1	5	6
3.9	18	2	4	6
7.8	16	6	3	9
0.125 MMC	24	32	26	58****
0.01 colchicine	3	18	19	37****

-S9 = in the absence of S9; +S9 = in the presence of S9; CP, cyclophosphamide; MMC, mitomycin C; SMLCT, short-, medium-, and long-chain triacylglycerides.

Significantly different from control group (Fisher’s exact test): *p < 0.05, **p < 0.01, ***p < 0.001, or ****p < 0.0001.

Duplicate cultures are indicated by A and B.

^a^ 1,000 binucleated cells were scored for the presence of micronuclei.

Based on the study criteria, SMLCT was concluded to be non-mutagenic under the conditions of the *in vitro* peripheral human lymphocyte micronucleus assay.

### 3.3 *In Vitro* mammalian gene mutation test in 5178Y mouse lymphoma cells

In the dose range–finding study, the test material was found to precipitate into the exposure medium at a concentration of 31 μg/mL or greater. However, there was no test substance–related toxicity reported in the presence or absence of metabolic activation at all tested concentrations relative to the negative control. Table 5 shows the percentages of cell survival and MFs for the various tested concentrations of SMLCT across different exposure times and metabolic activation conditions. The experiment met all acceptability criteria with the positive and negative controls remaining within the ranges of historical controls established by the testing laboratory. SMLCT did not induce any biologically relevant increases in MF in the 5178Y mouse lymphoma cell line, either in the presence or absence of metabolic activation.

**TABLE 5:** Cytotoxic and mutagenic testing of SMLCT in the mouse lymphoma L5178Y test system.

Dose (µg/mL)	RSG (%)	CE_Day2_ (%)	Relative cloning efficiency (%)	Relative total growth (%)	Mutation frequency per 10^6^ survivors		
					Total colonies	Small colonies	Large colonies
3-h treatment without metabolic activation							
Negative control –THF	100	78	100	100	143	95	41
5	95	84	107	102	152	94	49
7.5	130	93	119	154	152	88	54
10	136	79	101	138	125	83	37
12.5	130	78	100	130	95	49	43
15	122	54	69	84	222	137	72
20	130	81	104	136	127	76	45
25	118	91	117	138	96	58	34
31 ^a^	129	89	114	147	97	53	40
MMS	72	77	99	71	957	398	387
24-h treatment without metabolic activation							
Negative control – THF	100	113	100	100	110	34	70
Negative control – THF	-	99	-	-	106	31	70
0.25	104	116	109	114	90	24	63
0.5	87	123	116	101	108	27	75
1.0	71	139	130	93	93	32	55
2.0	85	129	121	103	102	39	57
3.9	83	111	105	87	99	26	69
7.8	74	104	98	72	125	34	84
16	71	91	86	61	148	38	103
31 ^a^	78	125	118	92	121	28	86
MMS	108	101	95	102	556	217	262
3-h treatment with metabolic activation							
Negative control – THF	100	95	100	100	124	58	59
Negative control – THF	-	80	-	-	150	91	51
5	113	64	73	83	174	71	93
7.5	111	84	95	106	113	49	59
10	124	93	106	131	132	59	64
12.5	131	81	93	121	145	75	62
15	118	67	77	91	152	82	63
20	123	81	93	114	123	76	41
25	115	79	90	103	138	59	71
31	106	88	100	105	135	54	74
CP	56	36	41	23	2533	1050	926

- = not reported; CE_Day2_ = cloning efficiency on Day 2; CP, cyclophosphamide; MMS, methyl methanesulfonate; RSG, relative suspension growth; SMLCT, short-, medium-, and long-chain triacylglycerides; THF, tetrahydrofuran.

^a^ The test material precipitated in the exposure medium.

Based on the study criteria, SMLCT was concluded to be non-mutagenic under the conditions of the *in vitro* 5178Y mouse lymphoma gene mutation test.

## 4 Discussion

Genetic toxicity testing is a valuable tool for evaluating the potential of substances to induce cellular events involved in the development of serious diseases, particularly cancer (Goodman et al., 2007). The potential genotoxicity and oral toxicity of various TAG preparations, alone or in combination with other TAGs, have been extensively evaluated in the peer-reviewed literature, and no findings indicating potential genotoxicity have been reported (Bach and Babayan, 1982; Traul et al., 2000; Matulka et al., 2009; Zhou et al., 2017; Jadhav and Annapure, 2021; Maki et al., 2021; Wang et al., 2023; Hartwig, 2024; Zhao et al., 2024). Mono- and polyunsaturated fatty acids within TAGs are highly susceptible to auto-oxidation and enzyme-catalyzed oxygenation. This process generates lipid hydroperoxides, 4-hydroxy-nonenal, and related α,β-unsaturated aldehydes that can form potentially mutagenic etheno- and propano-DNA adducts (Brown et al., 2019). The oxidation products of TAGs in palm oil, such as peroxides, epoxides, and unsaturated aldehydes, are reactive and have exhibited weak mutagenic potential *in vitro*; however, the addition of antioxidants, catalase, or S9 metabolic activation mitigated the reported mutagenic activity, confirming the indirect mechanism of action of oxidated damage rather than direct mutagenic effects on DNA (Kensese and Smith, 1989; Hartwig, 2024). Furthermore, co-administration of various plant-based oils with cisplatin in rats elicited protective effects against the genotoxicity of cisplatin in an *in vivo* chromosomal aberration test (Evangelista et al., 2006).

While the data reported in the literature do not suggest that SMLCT might be genotoxic, direct evaluation of genotoxic potential by validated methods is required for products intended for use in food. SMLCT is intended for use in food as a substitute for solid fat from animal or plant sources. The ratio of odd-chain to even-chain fatty acids in SMLCT is approximately 1:1, which is in sharp contrast with the 2:98 distribution of C15:0 and C17:0 relative to even-chain fatty acids reported for dairy-derived fat (Månsson, 2008). Although investigations into the safety and toxicity potential of several odd-chain fatty acid TAGs have been reported in the literature, SMLCT represents a novel composition of odd- and even-chain fatty acid TAGs and is therefore subject to evaluation to support a conclusion of safe use in food. The genotoxicity and mutagenicity potential of SMLCT was thus evaluated in a battery of standardized *in vitro* toxicity studies: a bacterial reverse mutation assay, an *in vitro* mammalian gene mutation assay, and an *in vitro* micronucleus assay in cultured human lymphocytes. The protocols were compliant with OECD Test Guidelines 471, 487, and 490, respectively (OECD, 2016; OECD, 2020; OECD, 2023). The *in vitro* reverse mutation assay primarily uses various strains of *S.* Typhimurium and *E. coli* to detect mutagenic effects, including those caused by frameshift and point mutations (Ames et al., 1975). The *in vitro* micronucleus assay in peripheral human lymphocytes is a more human-relevant model, as it uses eukaryotic cells. It is also employed to evaluate structural and numerical chromosomal alterations, such as chromatid breaks, chromosome exchanges, and aneuploidy, which reflect damage to chromosome integrity (Clare, 2012). The mouse lymphoma mutagenicity assay is used to investigate gene-level mutations at the TK locus in L5178Y cells and can detect point mutations, small intragenic deletions, and large chromosomal rearrangements. Analysis of colony size further distinguishes small colonies (indicative of large-scale chromosomal changes) from large colonies (which reflect point mutations), providing insight into the nature of the genetic lesions (Blazak et al., 1989). The test systems employed in this battery of genetic toxicity testing are complementary in assessing the potential of SMLCT to induce genotoxicity and mutagenicity.

The experiments reported herein, meeting the acceptance criteria detailed in the referenced guidelines, provide evidence that SMLCT is neither genotoxic nor mutagenic under the test conditions. The test concentrations used in the study were optimized based on the dose range–finding studies and in accordance with the respective test guidelines. As a saturated fat, SMLCT was insoluble in polar solvents such as water, and thus its solubility was tested in a range of solvents including acetone, hexane, ethanol, isopropyl alcohol, THF, and DMSO. Only in THF did the SMLCT fully dissolve into a clear solution (at 854 mg/mL), which remained stable for >24 h. However, due to the different test matrices employed in the assays, precipitate formation occurred, which limited the concentrations used in the studies.

The reported results demonstrate that SMLCT did not induce any genotoxic or mutagenic effects under the study conditions. SMLCT was not mutagenic in the bacterial reverse mutation assay at concentrations of up to 5000 µg/plate, non clastogenic or aneugenic at concentrations of up to 78.1 μg/mL in the *in vitro* micronucleus assay using peripheral human lymphocytes, and not mutagenic in the *in vitro* mouse lymphoma at a concentration of 31 mg/mL.

Regulatory evaluations of structurally related triacylglycerol-rich oils (e.g., medium-chain, eicosapentaenoic acid–rich, or structured triacylglycerols) consistently report negative findings in the standard battery of genotoxicity assays (bacterial reverse mutation, *in vitro* chromosomal aberration, and *in vivo* micronucleus tests). Similarly, many subchronic oral toxicity studies reported no-observed-adverse-effect levels at the highest doses administered, on the order of g/kg body weight/day, as would be expected for macronutrients lacking significant oral toxicity potential (Matulka et al., 2009; Zhou et al., 2017; Matulka et al., 2021; Wang et al., 2023; Zhao et al., 2024). These findings are similar to those reported for other dietary fat ingredients, which have tested negative in genetic toxicity studies including bacterial reverse mutation assays, *in vitro* mouse lymphoma gene mutation assays, *in vitro* chromosomal aberration assays, and unscheduled DNA synthesis tests in liver cells (Hayes and Riccio, 1994; Matulka et al., 2009; Matulka et al., 2021; Wang et al., 2023).

The absence of genetic toxicity in the studies reported herein contributes to the safety profile of the SMLCT synthetic fat ingredient.

## Data Availability

The original contributions presented in the study are included in the article/Supplementary Material, further inquiries can be directed to the corresponding author.
