# Supplementary material for: An In vitro assessment of the genotoxic potential of short-, medium-, and long-chain triacylglycerides
Source: Front Toxicol. 2026 Apr 8;8:1807786. doi: 10.3389/ftox.2026.1807786 (PMC13099124; doi:10.3389/ftox.2026.1807786)
Supplement: Supplementary file 1 [file Table1.docx]

Supplementary Material

# Supplementary Table

# Table S1. Dose range–‍finding study for the mutagenic response to SMLCT in the *Salmonella* Typhimurium mutation assay and the *Escherichia coli* mutation assay with or without metabolic activation

| **Concentration**  **(µg/plate)** | **Revertant Colonies per Plate (mean ± SD)** | | | | | | | | | | | | | |
| --- | --- | --- | --- | --- | --- | --- | --- | --- | --- | --- | --- | --- | --- | --- |
|  | ***Salmonella* Typhimurium** | | | | | | | | | | |  | ***Escherichia coli*** | |
|  | **TA98** | |  | **TA100** | |  | **TA1535** | |  | **TA1537** | |  | **WP2 *uvrA*** | |
|  | **-S9** | **+S9** |  | **-S9** | **+S9** |  | **-S9** | **+S9** |  | **-S9** | **+S9** |  | **-S9** | **+S9** |
| Vehicle control (THF) | 22.7 ±  8.4 | 26.7 ±  7.1 |  | 107.0 ±  23.4 | 93.7 ±  21.5 |  | 12.0 ±  1.0 | 7.3 ±  2.9 |  | 6.0 ±  4.4 | 6.3 ±  2.5 |  | 49.7 ±  10.1 | 56.7 ±  11.0 |
| 1.7 | 23.7 ±  7.6 | 34.0 ±  4.6 |  | 116.0 ±  16.5 | 80.7 ±  15.9 |  | 8.0 ±  2.0 | 11.3 ±  2.1 |  | 4.0 ±  3.0 | 8.3 ±  3.5 |  | 37.3 ±  5.5 | 49.0 ±  13.1 |
| 5.4 | 22.0 ±  7.9 | 29.0 ±  4.6 |  | 102.7 ±  12.0 | 100.0 ±  9.0 |  | 10.3 ±  3.1 | 12.0 ±  3.5 |  | 4.7 ±  2.9 | 4.0 ±  4.0 |  | 27.3 ±  3.8 | 47.7 ±  16.7 |
| 17 | 20.7 ±  3.1 | 26.7 ±  10.7 |  | 101.7 ±  19.9 | 82.0 ±  8.5 |  | 8.7 ±  1.2 | 14.7 ±  3.8 |  | 8.3 ±  3.2 | 6.3 ±  1.5 |  | 37.7 ±  2.5 | 39.3 ±  8.4 |
| 52 | 24.0 ±  9.0 | 32.0 ±  3.5 |  | 105.3 ±  4.7 | 89.3 ±  15.9 |  | 8.0 ±  3.6 | 14.7 ±  2.1 |  | 5.7 ±  2.1 | 7.0 ±  1.7 |  | 39.3 ±  11.0 | 53.0 ±  8.7 |
| 164 ^1^ | 19.3 ±  4.9 | 24.3 ±  2.5 |  | 105.0 ±  23.6 | 87.7 ±  13.6 |  | 10.7 ±  6.7 | 16.0 ±  6.1 |  | 8.3 ±  2.3 | 8.3 ±  5.8 |  | 29.7 ±  2.3 | 53.0 ±  8.5 |
| 512 ^1^ | 13.7 ±  4.5 | 33.7 ±  3.8 |  | 111.3 ±  14.2 | 98.3 ±  7.5 |  | 6.7 ±  1.2 | 8.7 ±  3.1 |  | 0.7 ±  0.6 | 7.0 ±  2.6 |  | 22.0 ±  7.0 | 51.0 ±  13.5 |
| 1600 ^1^ | 8.7 ±  3.1 | 16.0 ±  7.0 |  | 91.0 ±  13.0 | 75.3 ±  4.0 |  | 8.7 ±  2.9 | 6.0 ±  4.4 |  | 4.3 ±  0.6 | 5.3 ±  3.2 |  | 22.0 ±  4.6 | 43.3 ±  5.7 |
| 5000 ^1^ | 11.7 ±  1.5 | 17.7 ±  3.2 |  | 82.0 ±  23.1 | 62.7 ±  14.2 |  | 6.7 ±  3.2 | 8.7 ±  2.1 |  | 1.3 ±  0.6 | 2.7 ±  0.6 |  | 21.3 ±  0.6 | 30.0 ±  7.9 |
| Positive control ^2,3^ | 1900.7 ±  145.1 | 2232.3 ±  484.5 |  | 1024.0 ±  98.0 | 2180.3 ±  126.4 |  | 1065.3 ±  137.2 | 234.0 ±  39.3 |  | 1151.0 ±  60.0 | 226.7 ±  76.7 |  | 1781.0 ±  103.5 | 218.7 ±  86.4 |

-S9 = in the absence of S9; +S9 = in the presence of S9; 2‑AA = 2-aminoanthracene; 2-NF = 2-nitrofluorene; 4‑NQO = 4-nitroquinoline *N*-oxide; AAC = 9-aminoacridine hydrochloride; ICR‑191 = acridine mutagen ICR‑191; MMS = methyl methanesulfonate; SA = sodium azide; SD = standard deviation; SMLCT = short-, medium-, and long-chain triacylglycerides; THF = tetrahydrofuran.
^1^ The test material precipitated in the exposure medium.

^2^ Positive control -S9: TA98 = 10 µg/plate 2-NF; TA100 = 650 µg/plate MMS; TA1535 = 5 µg/plate SA; TA1537 = 2.5 µg/plate ICR-191; WP2 *uvrA* = 10 µg/plate 4‑NQO.

^3^ Positive control +S9: TA98 = 1 µg/plate 2‑AA; TA100 = 2 µg/plate 2‑AA; TA1535 = 2.5 µg/plate 2‑AA; TA1537 = 5 µg/plate AAN; WP2 *uvrA* = 15 µg/plate 2‑AA.
